# Does high intensity exercise affects irisin plasma levels in hemodialysis patients? A pilot study

**DOI:** 10.1590/1678-4685-JBN-3802

**Published:** 2018-04-23

**Authors:** Marta Gormicho Boavida Marques Esgalhado, Milena Barcza Stockler-Pinto, Ludmila Ferreira Medeiros de França Cardozo, Jorge Eduardo Barboza, Denise Mafra

**Affiliations:** 1 Universidade Federal Fluminense, Programa de Pós-Graduação em Ciências Cardiovasculares Niterói, Rio de Janeiro (RJ), Brasil.; 2 Universidade Federal Fluminense, Programa de Pós-Graduação em Ciências Médicas Niterói, Rio de Janeiro (RJ), Brasil.

**Keywords:** Resistance Training, Renal Dialysis, Hormones, Treinamento de Resistência, Diálise Renal, Hormonas

## Abstract

**Background:**

Irisin is a recently identified exercise-induced hormone that stimulates the "browning" of the white adipose tissue, at least in mice. In chronic kidney disease (CKD) patients, irisin regulation is not fully understood, and little attention has been given to the effects of exercise on irisin levels in these patients. The purpose of this study was to assess the effects of high intensity exercise on irisin plasma levels in CKD patients under hemodialysis (HD).

**Methods:**

Fifteen HD patients (5 men, 44.4 ± 15.1 years old) were studied and served as their own controls. High intensity (single session) intradialytic strength exercises consisted of three sets of ten repetitions with four different movements in both lower limbs during 30 minutes. Blood samples were collected on different days (exercise and non-exercise day) at exactly the same time (30 and 60 minutes after the start of dialysis session). Plasma irisin levels were measured by ELISA assay and anthropometric and biochemical parameters were evaluated.

**Results:**

Irisin plasma levels were significantly reduced in both exercise day (125.0 ± 18.5 to 117.4 ± 15.0 ng/mL, *p*=0.02) and non-exercise day (121.5 ± 13.7 to 115.4 ± 17.2 ng/mL, *p*=0.02) after 60 minutes of dialysis.

**Conclusion:**

These data suggest that intense intradialytic strength exercise was unable to increase the circulating concentration of irisin in HD patients. Moreover, our data show that after one hour of dialysis session, irisin plasma levels may be reduced.

## RESUMO

**História:**

A irisina é um hormônio induzido pelo exercício recentemente identificado que estimula o "escurecimento" do tecido adiposo branco, pelo menos em camundongos. Nos pacientes com doença renal crônica (DRC), a regulação da irisina não é totalmente compreendida, e pouca atenção tem sido dada aos efeitos do exercício sobre os níveis de irisina nesses pacientes. O objetivo deste estudo foi avaliar os efeitos do exercício de alta intensidade sobre os níveis plasmáticos de irisina em pacientes com DRC em hemodiálise (HD).

**Métodos:**

15 pacientes em HD (5 homens, 44,4 ± 15,1 anos) foram estudados e serviram como os próprios controles. Os exercícios de resistência intradialítica de alta intensidade (sessão única) consistiram em três séries de dez repetições com quatro movimentos diferentes em ambos os membros inferiores durante 30 minutos. As amostras de sangue foram coletadas em dias diferentes (dia de exercício e dia sem exercício) exatamente no mesmo horário (30 e 60 minutos após o início da sessão de diálise). Os níveis de irisina plasmática foram medidos por ensaio ELISA e os parâmetros antropométricos e bioquímicos foram avaliados.

**Resultados:**

Os níveis plasmáticos de irisina foram significativamente reduzidos tanto nos dias de exercício (125,0 ± 18,5 a 117,4 ± 15,0 ng/mL, *p*=0,02) quanto nos dias sem exercício (121,5 ± 13,7 a 115,4 ± 17,2 ng / mL, *p*=0,02), após 60 minutos de diálise.

**Conclusão:**

esses dados sugerem que o exercício intenso de resistência intradialítica não aumentou a concentração circulante de irisina em pacientes sob HD. Além disso, nossos dados mostram que após uma hora de sessão de diálise, os níveis plasmáticos de irisina podem ser reduzidos.

## INTRODUCTION

It is well known that physical exercise is effective in improving body composition and metabolic health; however, the underlying mechanisms for its clinical benefits are still unclear.^1^^,^ 2 Some of the best-recognized effects of exercise on muscle are mediated by the peroxisome proliferator-activated receptor γ co-activator-1α (PGC-1α ) that stimulates the expression of the transmembrane protein fibronectin type III domain containing 5 (FNDC5). The FNDC5 seems to undergo a proteolytic cleavage, releasing a small fragment called irisin (the remaining 112 amino acid residues) into the blood.^3^ Irisin is a hormone that can bind to undetermined receptors on the surface of cells in adipose tissue, stimulating its "browning" and thermogenesis in mice by increasing uncoupling protein 1 (UCP-1) expression.^3^ Therefore, irisin can convert white adipose tissue into "beige adipose tissue", which is associated to positive effects on health, such as fat mass control, glucose tolerance and insulin resistance improvement, prevention of muscle loss, and reduction of systemic inflammation.^3^^-^ 5 Thus, irisin has gained great interest as a potential new target for preventing and treating metabolic disorders like obesity and diabetes.^1^^,^ 3 ^,^ 6 ^,^ 7 Nonetheless, some controversies have arisen among recent publications regarding the importance of irisin and/or FNDC5 in humans.^1^^,^ 4 ^,^ 8 ^,^ 9 Probably, most disagreements might be due to different exercises, their duration, the training status of individuals, and the type of assay for irisin quantification.^10^

Several researchers have examined the effects of physical exercise on irisin levels in healthy individuals.^3^^-^ 5 ^,^ 11 ^-^ 19 Huh et al.^19^ observed higher irisin plasma levels in young males after 30 minutes of sprint exercise but not after 8 weeks of training intervention. Nygaard et al.^5^ revealed that single sessions of intense endurance exercises and heavy strength training led to transient increases in irisin levels in healthy subjects.

Irisin levels have not been comprehensively studied in patients with chronic diseases like obesity, diabetes, and chronic kidney disease (CKD) and little attention has been given to the effects of exercise on irisin levels in these patients. A study conducted in pre-diabetes subjects showed that 12 weeks of training reduced circulating irisin and, in contrast, acute exercise increased its levels (~1.2-fold). Moreover, plasma irisin levels were higher in pre-diabetes subjects compared with controls.^20^ Corroborating these facts, Moraes et al.^21^, observed that 6 months of training did not increase plasma irisin levels in CKD patients undergoing hemodialysis (HD). Following this line of thought, it is suggested that a single session (intense) exercise would increase irisin plasma levels in these patients.

Despite the increase of irisin plasma levels in healthy^5^^,^ 12 ^-^ 14 ^,^ 19 and pre-diabetic subjects^20^ after a single session of exercise, the acute effects of exercise in CKD patients are still unknown. The aim of this study was to assess the effect of high intensity exercise on irisin plasma levels in CKD patients on HD.

## METHODS

### RECRUITMENT OF PARTICIPANTS

Fifteen eligible CKD patients undergoing HD were recruited among patients from the Renal Vida Clinic in Rio de Janeiro, Brazil, previously included in an investigation from our research group.^22^ Inclusion criteria were as follows: older than 18 years, dialysis treatment for at least 6 months before the study, using biocompatible membranes (low-flux polysulfone), with arteriovenous fistula for vascular access in the upper limb, and absence of motor skill disorders and ability to perform strength physical exercise. Patients with autoimmune, infectious, inflammatory, neurological, cardiovascular, lung or neoplastic diseases, HIV, uncontrolled hypertension, malignant arrhythmias, unstable angina, history of stroke (either ischemic or hemorrhagic) were excluded as well as patients on acute dialysis, smokers, lower limb amputees, patients that were hospitalized within 3 months before the study and pregnant women. Patients who regularly practiced physical exercise were also excluded. Patients served as their own control on a non-exercise day.

### ETHICAL CONSIDERATIONS

All procedures complied with the principles of the Declaration of Helsinki. The study protocol was approved by the local Ethics Committee (Medical Faculty of Federal Fluminense University) with the project number 301/11. All patients provided written informed consent to participate in the study. This study was registered in the Clinical Trials service of the US National Institutes of Health (clinicaltrials.gov), under the follow identification number: NCT02718638.

### INTERVENTION: PHYSICAL EXERCISE PROGRAM

The exercise program was handled by a physical therapist and consisted of three sets of ten repetitions with four different movements with ankle cuffs and elastic bands (Theraband, Akron, OH, USA) in both lower limbs, as previously described:^22^

1) Knee extension: from 90º to 0º, for isometric contraction the patient remained in the 0º position for 5 seconds then returned to the 90º position;

2) Triple flexion followed by extension of the lower limbs: An elastic band was placed at the level of the metacarpals and the patient flexed the thigh, knee, and ankle muscles, followed by a double extension of the thigh and knee muscles;

3) Co-isometric contraction: With an ankle-cuff resistance band located under the distal third of the leg at the level of the malleolus, the patient performed a leg extension for 10 seconds;

4) Unilateral hip joint flexion: Patients were asked to extend the knee by rising to their functional limit.

Patients performed ten repetitions of each exercise and rested 3 minutes between the four exercise categories. The single session of exercises was performed during 30 minutes in the second half-hour of the dialysis session. The intensity was based on adaptation of 1-repetition maximum test and the initial intensity was 60%, once most CKD patients are debilitated.

### NUTRITIONAL ASSESSMENT

The body mass index (BMI) was calculated as the dry body weight (kg) divided by the squared height (m) and was used to assess the patient's nutritional status according to the World Health Organization.^23^ Body fat was calculated from skinfold measurement (mm) (at four standard sites (biceps, triceps, subscapular, and suprailiac) using a Lange Skinfold Caliper (Cambridge Scientific Products(®), Cambridge, MA, USA)) according to Durnin & Womersley's equation,^24^ and the % body fat was calculated using Siri's equation.^25^ The arm muscle area (AMA) was calculated according to the Heymsfield equation.^26^ All measurements were performed after the dialysis session by a trained staff member.

### BIOCHEMICAL ANALYSES

Fasting blood samples were collected in the morning on two different days, one in a day without exercise and one in a day with a bout of intradialytic strength exercise, at exactly the same time (30 and 60 minutes after initiating the dialysis session). Samples were centrifuged (15 minutes, 3500 rpm, 4ºC) and stored in tubes at -80ºC until analysis. Data collection was done 30 minutes after initiating dialysis session to allow patients to adapt to the dialysis process. As the physical exercise duration was 30 minutes, we collected blood samples 60 minutes after starting dialysis session, i.e., immediately after the exercise session and not after HD session of 4 hours.

Like in the previous study, routine biochemical parameters (K, P, albumin, hematocrit, hemoglobin, glucose, and urea pre and post-dialysis) were measured according to standard methods. Serum levels of high sensitivity C-reactive protein (hs-CRP) were analyzed using Bioclin(®) kits (Catalog #K079) by automatic biochemical analyzer. Kinetic index of dialysis adequacy (Kt/V) was calculated according to Daugirdas equation.^27^

Plasma irisin levels were assessed using an enzyme-linked immunosorbent assay (ELISA) from a commercial kit according to the manufacturer's instructions (Phoenix Pharmaceuticals, Burlingame, CA, USA).

### STATISTICAL ANALYSIS

The distribution of the variables was analyzed by Shapiro-Wilk test. The results are expressed as the mean ± SD (standard deviation) or percentages, as applicable. A paired student's t-test was used to compare the variables. The correlations between variables were analyzed using Pearson correlations coefficient. The values obtained after exercise were compared with their own controls. Statistical analyses were performed using SPSS 19.0 software (Chicago, IL, USA).

## RESULTS

Fifteen HD patients (5 men, 44.4 ± 15.1 years) with dialysis vintage of 64.9 (19.5 - 94.5) months were studied. The main etiology for CKD was hypertension (73%) followed by chronic glomerulonephritis (13%) and others diseases (14%). Anthropometric and biochemical baseline parameters are shown in the Table 1. According to BMI, only 2 patients presented values below 18.5 Kg/m^2^ and 3 were overweight/obese; all patients presented albumin levels higher than 3.8 mg/dL.

**Table 1:** Baseline anthropometric and biochemical characteristics of the enrolled patients

Parameters	Values
Body mass index (kg/m^2^)	23.4 ± 5.1
% fat mass	31.5 ± 8.2
Arm muscle area (cm^2^)	32.1 ± 12.9
Potassium (mg/dL)	4.7 ± 0.7
Phosphorus (mg/dL)	6.9 ± 1.7
Albumin (mg/dL)	4.1 ± 0.2
Hematocrit (%)	30.9 ± 5.2
Hemoglobin (g/dL)	10.2 ± 1.7
Glucose (mg/dL)	86.7 ± 19.5
Dialysis dose - Kt/V	1.57 ± 0.4

Plasma irisin levels revealed no significant difference between non-exercise and exercise days at baseline. In contrast, irisin levels were significantly reduced after 60 minutes of HD session in both moments: with 30 minutes of intradialytic strength exercise, from 125.0 ± 18.5 to 117.4 ± 15.0 ng/mL (*p*=0.02) and without exercise session, from 121.5 ± 13.7 to 115.4 ± 17.2 ng/mL, (*p*=0.02). Moreover, irisin levels did not differ according to gender. The hs-CRP levels did not change after 60 minutes of HD session in both moments. Furthermore, a bivariate correlation analysis revealed that circulating irisin was negatively correlated with dialysis vintage (r = -0.54, *p* = 0.03); there was no correlation with anthropometric data or with biochemical parameters.

## DISCUSSION

The present study showed that intense acute intradialytic strength exercise was unable to increase plasma irisin levels in HD patients. Irisin plasma levels were reduced after a one-hour HD session. Irisin is a hormone secreted during exercise that increases energy expenditure by stimulating the expression of UCP-1 and, thus, the "browning" of white adipose tissue.^3^ This novel hormone has been proposed to be an attractive future therapeutic target for metabolic disorders.^1^^,^ 3 ^,^ 6 ^,^ 7 However, the effects of irisin in humans are still unclear and, data regarding circulating irisin levels in patients with CKD are still lacking.

Studies have observed an increase in irisin plasma levels after chronic exercise;^3^^,^ 15 however, some authors have reported that a single session of exercise, but not chronic exercise, promotes an increase in circulating irisin.^12^^-^ 14 ^,^ 19 ^,^ 20

The study conducted by Huh et al.^13^ observed that whole-body vibration exercise for 1.5 months in healthy untrained females did not change irisin levels, but an intense exercise was effective. Kraemer et al.^1^ reported an increase in irisin levels after 54 minutes of treadmill exercise in healthy young individuals, but by 90 minutes of exercise, levels were no longer elevated. Moreover, in inactive pre-diabetes individuals, circulating irisin levels were reduced in response to 12 weeks of training, and was acutely increased just after intense exercise.^20^

Resistance exercise seems to be more effective in increasing irisin levels than endurance exercise.^4^^,^ 11 Nonetheless, some studies found no change in irisin levels after acute, chronic, endurance or resistance exercise was observed.^9^^,^ 16 ^-^ 18 Thus, we are still unaware of which exercise type, intensity or duration, if any, would be effective in increasing plasma irisin levels in the general population.

Our previous study revealed that a resistance exercise training program for 6 months was unable to increase plasma irisin levels in HD patients.^21^ According to the present study, intense acute intradialytic strength exercise also seemed ineffective for the increase of circulating concentration of irisin in HD patients. There are two possible explanations for our results. Firstly, the intervention was too short, not allowing the mobilization of irisin from its precursor or the FNDC5 to be recruited to the membrane. Secondly, we measured irisin concentrations immediately after the intervention, but their elevation may occur minutes or hours after the end of the physical exercise session.

Circulating irisin concentration is decreased with increasing CKD stage.^28^^-^ 30 The mechanism underlying this decreased is still unknown. One possible explanation can be the negative correlation between blood urea nitrogen and decreased irisin levels, showing good evidence on how uremia may affect irisin levels.^30^ Additionally, CKD patients might have lower muscle volume, and irisin, which is produced within muscle, can be affected by total muscle volume.^31^ In this study, we did not find a correlation between plasma irisin levels and anthropometric data.

Furthermore, after an hour of dialysis we observed a decrease in irisin levels in both moments (with and without exercise). This is the first evidence that plasma irisin decreased with HD session. Irisin is a peptide with 112 amino acids and ~12 kDa molecular weight^32^ and because of its molecular weight, irisin does not easily pass through the pores of the dialysis membrane (though it might pass sporadically).^33^ One possible explanation is that irisin is absorbed by the polysulfone membrane. Thus, if plasma irisin levels were not naturally reduced by the HD process, they might have increased with this exercise.

The clinical relevance of irisin in humans was outside the scope of this study; nevertheless, this topic needs to be further explored. According to Nygaard et al.^5^ physical exercise (endurance, strength training or both) does not increase steady-state irisin levels, but it may have cumulative health effects, because of transient increases in circulating irisin produced during physical exercise.

Some limitations should be taken into consideration when interpreting our results. First, the relatively small sample size, intrapatient variability, and limited data collected from the patients should be considered. Second, we did not collect blood samples (hourly) 4 hours post-exercise to evaluate the modulation of plasma irisin levels. Considering that PGC-1α is up-regulated 2-3hours after exercise, irisin concentration may be maximized at 2hours following exercise.^4^ Lastly, there are controversies about the reliability of ELISA kits for detecting serum irisin, so we suggest adding an alternative assay, like mass spectrometry analysis or western blotting to avoid any cross-reacting proteins. The present study used the most prevalent ELISA kit (Phoenix Pharmaceuticals) in accordance with some of the previous studies.^11^^,^ 12 ^,^ 15 ^,^ 20 ^,^ 29 ^,^ 30 This kit has been previously validated against western blotting^11^^,^ 25 and mass spectrometry,^34^ with a detection range between 0.1 and 1000 ng/mL. In spite of these limitations, this was a very well controlled protocol, which allowed us to conclude that the results are considerably relevant.

In summary, these data suggest that acute intradialytic strength exercise was unable to increase the circulating concentration of irisin in CKD patients undergoing HD. Additionally, it seems that the dialysis process itself may reduce irisin plasma levels. Thus, considering the important benefits of physical exercise and the scarcity of information on this subject, further studies with the same or an alternative exercise modality, intensity or time, are needed to evaluate the regulation of plasma irisin and its physiological effects in CKD patients (mainly related to metabolic balance).

### PRACTICAL APPLICATION

Irisin is an exercise-induced myokine known for stimulating the "browning" and thermogenesis of adipose tissue and, therefore, is involved in fat mass control, glucose tolerance and insulin resistance improvement, prevention of muscle loss, and reduction of systemic inflammation. CKD is characterized by altered energy expenditure and metabolism. In this context, irisin may be a promising therapeutic agent for preventing CKD development and complications. However, the effects of irisin in CKD patients are still unclear. Considering the importance and the scarcity of information on this subject, further studies are needed.
